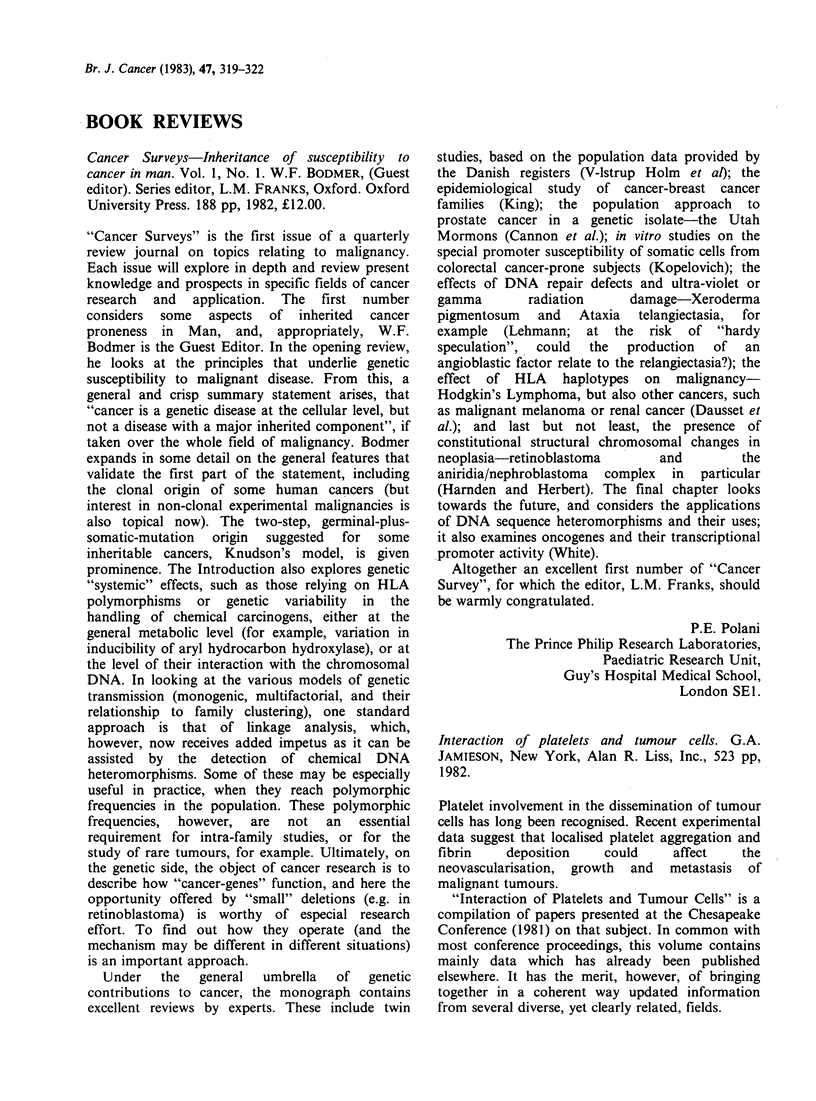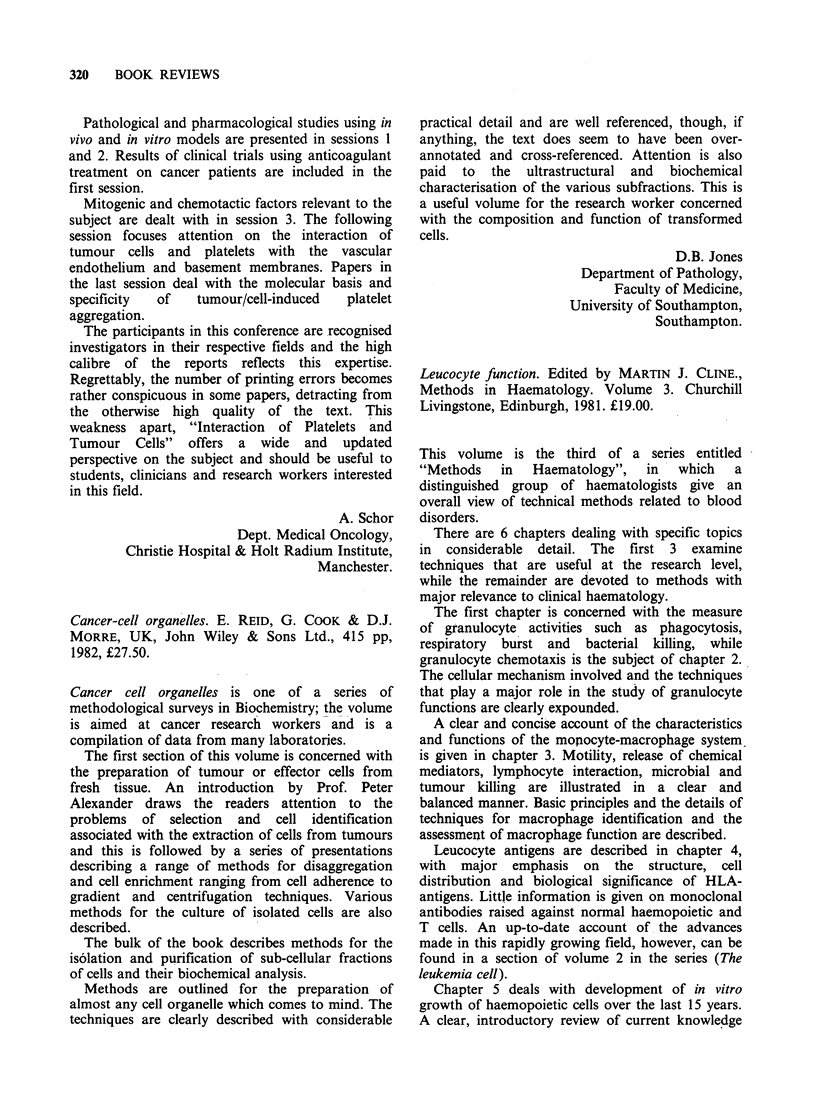# Interaction of platelets and tumour cells

**Published:** 1983-02

**Authors:** A. Schor


*Interaction of platelets and tumour cells.* G.A. JAMIESON, New York, Alan R. Liss, Inc., 523 pp, 1982.

Platelet involvement in the dissemination of tumour cells has long been recognised. Recent experimental data suggest that localised platelet aggregation and fibrin deposition could affect the neovascularisation, growth and metastasis of malignant tumours.

"Interaction of Platelets and Tumour Cells" is a compilation of papers presented at the Chesapeake Conference (1981) on that subject. In common with most conference proceedings, this volume contains mainly data which has already been published elsewhere. It has the merit, however, of bringing together in a coherent way updated information from several diverse, yet clearly related, fields.

Pathological and pharmacological studies using *in vivo* and *in vitro* models are presented in sessions 1 and 2. Results of clinical trials using anticoagulant treatment on cancer patients are included in the first session.

Mitogenic and chemotactic factors relevant to the subject are dealt with in session 3. The following session focuses attention on the interaction of tumour cells and platelets with the vascular endothelium and basement membranes. Papers in the last session deal with the molecular basis and specificity of tumour/cell-induced platelet aggregation.

The participants in this conference are recognised investigators in their respective fields and the high calibre of the reports reflects this expertise. Regrettably, the number of printing errors becomes rather conspicuous in some papers, detracting from the otherwise high quality of the text. This weakness apart, "Interaction of Platelets and Tumour Cells" offers a wide and updated perspective on the subject and should be useful to students, clinicians and research workers interested in this field.

A. Schor
Dept. Medical Oncology,
Christie Hospital & Holt Radium Institute,
Manchester.